# *In vitro* protein digestibility to replace *in vivo* digestibility for purposes of nutrient content claim substantiation in North America's context

**DOI:** 10.3389/fnut.2024.1390146

**Published:** 2024-05-24

**Authors:** Elaine S. Krul, Amanda G. A. Sá, Erin M. Goldberg, James D. House

**Affiliations:** ^1^EKSci, LLC, St. Louis, MO, United States; ^2^Richardson Centre for Food Technology and Research, Winnipeg, MB, Canada; ^3^Department of Food and Human Nutritional Sciences, University of Manitoba, Winnipeg, MB, Canada

**Keywords:** protein quality, nutrient content claims, *in vitro* protein digestibility, regulatory testing, food labeling

## Abstract

The reliance by North American regulatory authorities on *in vivo* rodent bioassays—Protein Correct-Amino Acid Score (PDCAAS) in the U.S. and Protein Efficiency Ratio (PER) in Canada—to measure the protein quality for protein content claim substantiation represents a major barrier for innovation in the development and marketing of protein foods. Although FAO in 2013 proposed a new method (Digestible Indispensable Amino Acid Score, DIAAS), it is still not used for protein content claim substantiation in any jurisdiction. Together with public health efforts to increase the consumption of plant-based foods, removing hurdles is key to incentivizing the food industry to measure protein digestibility in making food formulation decisions as well as in claiming protein content on product labels. To address this issue, a pathway has been proposed to position alternative methods for *in vitro* protein digestibility in collaborative studies to generate the data necessary for method approval by a certifying body. The latter is critical to the potential recognition of these methods by both Health Canada and the US FDA. The purpose of this article is to briefly summarize the state-of-the-art in the field, to inform the research community of next steps, and to describe the path engaging collaborative laboratories in a proficiency test as the first step in moving forward toward acceptance of *in vitro* digestibility methods. Throughout, a consultative and iterative process will be utilized to ensure the program goals are met. Success will be achieved when the proposed path results in the acceptance of an *in vitro* methods for protein digestibility used for PDCAAS determinations, which will enable increased protein analyses and improved nutrition labeling of protein foods.

## 1 Introduction

The definition of protein quality has historically been based on the ability of dietary protein to provide sufficient levels of indispensable (essential) amino acids to meet the metabolic requirements of humans (1, 2). This property is dependent on the protein's amino acid composition and digestibility/availability (3, 4). Protein content claims in the USA and Canada (but not in many other countries) for consumer foods not intended for special medical uses require the use of *in vivo* animal models to assess the quality of the protein (5, 6). In the US, the Food and Drug Administration (FDA) requires the determination of the protein digestibility-corrected amino acid score (PDCAAS) for protein content claim substantiation (7). The PDCAAS is determined as the product of the amino acid score and the true fecal protein digestibility (TFPD) of the test article in question (2). The amino acid score is determined by dividing the indispensable amino acid composition of the test article by the corresponding reference amino acid requirement values (mg/g protein) (8), with the score established as the lowest ratio value. As such, the AAS is a value that requires only analytical chemistry techniques or published amino acid composition tables for its calculation. However, the second component of the PDCAAS, the true fecal protein digestibility coefficient, requires the use of a rodent bioassay for its determination and represents an estimate of the extent to which the food protein is digested and absorbed. The use of a bioassay to assess protein quality is not unique to the PDCAAS as, in Canada, the protein rating system for content claim substantiation is typically based on the use of the protein efficiency ratio (PER) bioassay (9). The latter compares the growth rate of rats fed test protein compared to casein. The use of PER for general food labeling is unique to Canada and has been reviewed elsewhere (5, 6, 10). In December 2020, Health Canada announced that it would allow the usage of PDCAAS for the calculation of a Protein Rating (PER = PDCAAS × 2.5) for protein content claim substantiation, allowing this method to be harmonized between Canadian and American regulators and food industry stakeholders (11).

It is important to note that international organizations have convened expert panels on numerous occasions to assess measures of protein quality (8). Following a meeting in 2011 in Auckland, New Zealand, an expert panel prepared a document that positioned a refined method for determining the quality of dietary proteins, namely the Digestible Indispensable Amino Acid Score (DIAAS) method (12). Conceptually similar to PDCAAS, DIAAS relies on the use of updated amino acid reference patterns as well as an alternative strategy to assess the utilization of dietary protein. For the latter, the positioned method relies on the assessment of the ileal digestibility of dietary amino acids, instead of total nitrogen/crude protein. Finally, while the optimal subjects for study are humans, for practical purposes, the use of an ileal cannulated swine model has become the standard for the generation of DIAAS data on numerous food products (13, 14). While the DIAAS method has documented advantages over PER and PDCAAS, to date neither Health Canada nor the FDA have indicated an intention to move to this approach for protein content claim substantiation, with PDCAAS remaining the method of choice. This was recently reinforced by Health Canada when, in 2023, they signaled their intent to move even further with the adoption of PDCAAS for protein claims (15).

As reviewed by FAO/WHO (8, 12, 16), there are multiple methods for assessing protein quality, and the literature presents an exhaustive summary of these methods (1, 2, 17). The goal of this current work is to review the challenges that exist for the protein food sector in measuring PDCAAS values as currently required by regulatory authorities, with the primary concern relating to the use of rodent bioassays for measuring true fecal protein digestibility (TFPD). Another purpose of this work is to inform the research community of the options for replacing the TFPD value with those determined by suitable *in vitro* assays with an ongoing program for method validation with the first step being an interlaboratory collaborative study. A line of reasoning supporting the use of *in vitro* versus animal testing for protein quality assessment in North America has been positioned in a recent publication (18).

## 2 Determination of true fecal protein digestibility

According to Title 21 in the U.S. Code of Federal Regulations, the official method for evaluating protein digestibility for PDCAAS determination is the TFPD method. This method is outlined by the Food and Agricultural Organization/World Health Organization (FAO/WHO) in 1991, originally positioned by McDonough et al. (19), and is mandated by the U.S. Food and Drug Administration. The TFPD (%) of protein is determined as follows:


$$\begin{array}{l} TFPD\;\left(\%\right)\;=\;\frac{N_{I}-\left(F_{N}-M_{N}\right)}{N_{I}}\times 100 \end{array}$$

where *N*_*I*_ = N (nitrogen) intake (protein in diet), *F*_*N*_ = fecal N and *M*_*N*_ = fecal metabolic loss. *M*_*N*_ is determined from N measured in feces from rats fed protein-free diets. Minimally, this bioassay requires a minimum of four rats per test group (including a protein-free group for metabolic N losses) and 9 days to complete. As such, the TFPD measures the proportion of protein-derived nitrogen that is available (digestible) to meet the protein needs of the respective consumer. As positioned above, the TFPD is used to calculate the final PDCAAS value for a food or food ingredient.

With respect to the measurement of TFPD, the rat fecal balance method is relatively straightforward, and requires processes to ensure adequate measurement of feed intake, total fecal collection, including the use of wire bottomed cages, and feed and fecal nitrogen determinations. However, recognition that rodent nutrient requirements differ from human, that the large intestine microbiota can alter the amino acid composition of the digesta (20) and changes in laboratory animal welfare policies since this method was first positioned, create challenges for the current usage of this method. Although the FDA and Health Canada still requires the use of rodent models for protein quality determination and substantiation of protein content claims, the FDA Modernization Act 2.0 (Bill S.2952) was passed in the USA Congress in 2022, which removes the obligation for pharmaceutical companies to test drugs on animals before human trials. Societal expectations regarding the use of animal testing for regulatory purposes have also evolved. In response to strong external pressures, many food and ingredient companies have adopted policies against the use of animals in research and testing. Additionally, certain third-party validation and front-of-pack labeling systems that provide information to prospective consumers on the nature of the food (e.g., Certified Vegan™; Vegan Action/Vegan Awareness Foundation, 2021) stipulates that animals cannot be used in testing. This can place certain desirable and informative logos out of reach of the food sector, such as “good or excellent protein source” claims. Furthermore, there are global efforts by researchers and government agencies to replace, reduce, and refine (3Rs) the use of research animals in the safety evaluation of consumer goods, pharmaceuticals, and agricultural and industrial chemicals (21–23). Finally, developing validated *in vitro* methods for assessing protein digestibility will not only enable higher throughput of protein digestibility determinations, but will eliminate the high research costs associated with purchasing lab animals, maintaining animal housing facilities and formulating diets containing the appropriate protein test articles.

In Europe, the United States and Canada, all proposed animal research is reviewed by an appropriate organizational animal welfare committee to justify the use of animals and demonstrate how efforts have been made to comply with the 3Rs (24, 25). The “new approach methodologies” (NAMS) can be applied to this purpose. The NAMS term refers to any non-animal technology, methodology, approach, or combination thereof that can increase testing capacity as a result of significant advances in *in silico, in chemico* and *in vitro* method development (26). Completely animal-free methods, which do not use any animal component, are defined as “non-animal methods,” whereas replacement methods may still be dependent on animal components such as serum or enzymes (22). When NAMS are to be applied for regulatory purposes, it is important that assay developers and regulators work together to achieve agreement for adequate performance criteria for the specified context of use (26, 27). This holds true for the positioning of alternative, *in vitro* methods for the measurement of protein digestibility for the ultimate calculation of PDCAAS values.

## 3 *In vitro* protein digestibility assays

Current *in vitro* models used for the evaluation of protein digestibility include the use of both “dynamic” and “static” methods (28). Dynamic gastrointestinal digestion methods are designed to simulate gastro-intestinal digestion phases and nutrient bioaccessibility through the use of sophisticated, computer-controlled, temperature-regulated digestion chambers (28). These models have been used to measure amino acid and nitrogen digestibility (29), but the high cost of system acquisition, limited sample throughput, and high operational costs likely represent major limitations to their routine use for the systematic determination of protein or amino acid digestibility. However, dynamic models offer useful tools for the integrative study of nutrient digestion.

In comparison to dynamic model systems, static models represent “bench top” assays that can be readily implemented across various laboratory settings. Static *in vitro* assays that treat suspensions of food with a mix of digestive enzymes have been used for decades as research tools to study food structure and digestibility, nutrient bioavailability and to provide protein digestibility coefficients (30, 31). Many studies have reported PDCAAS values based on static *in vitro* digestibility for dozens of protein foods (1, 32–38). Static *in vitro* protein digestibility methods have fewer ethical concerns, are less costly, and can be executed more easily and rapidly and with much higher throughput than *in vivo* methods. This would enable a wider variety of raw and processed foods to undergo analyses for protein quality than is possible with the currently approved methods. While *in vitro* methods may not perfectly replicate *in vivo* digestibility, there is good agreement for digestibility values obtained with the two methods (1, 32–39). Recent summative data from the authors' laboratory provide evidence of high *R*^2^ values for PDCAAS values of plant-based protein sources when comparing those calculated via *in vivo* and *in vitro* assays. Sá et al. (40) reviewed various studies suggesting strong correlations between *in vitro* protein digestibility values (performed by pH-drop method) and *in vivo* protein quality measurements (e.g., PDCAAS) for different pulses: green and red lentils (*R*^2^ = 0.9971) (36), chickpea (*R*^2^ = 0.9442) (37), beans (*R*^2^ = 0.7497) (35), and pinto bean (*R*^2^ = 0.9280) (34). Furthermore, another study compared *in vitro* and *in vivo* PDCAAS for protein isolates and concentrates from faba beans, lentils, and peas, and the results showed a strong correlation (*R*^2^ = 0.9898) (41). Evidence shows that despite the simplicity of *in vitro* models (42), they are often very useful in predicting outcomes of the *in vivo* digestion (43). Thus, *in vitro* protein digestibility could be applied as a surrogate to calculate *in vitro* PDCAAS for determining protein quality. In considering static *in vitro* digestibility models, the methods can vary in complexity, from simple mono-compartmental models to those that simulate multiple gastrointestinal compartments.

### 3.1 Static, mono-compartmental models for determining protein digestibility

**Figure 1:**
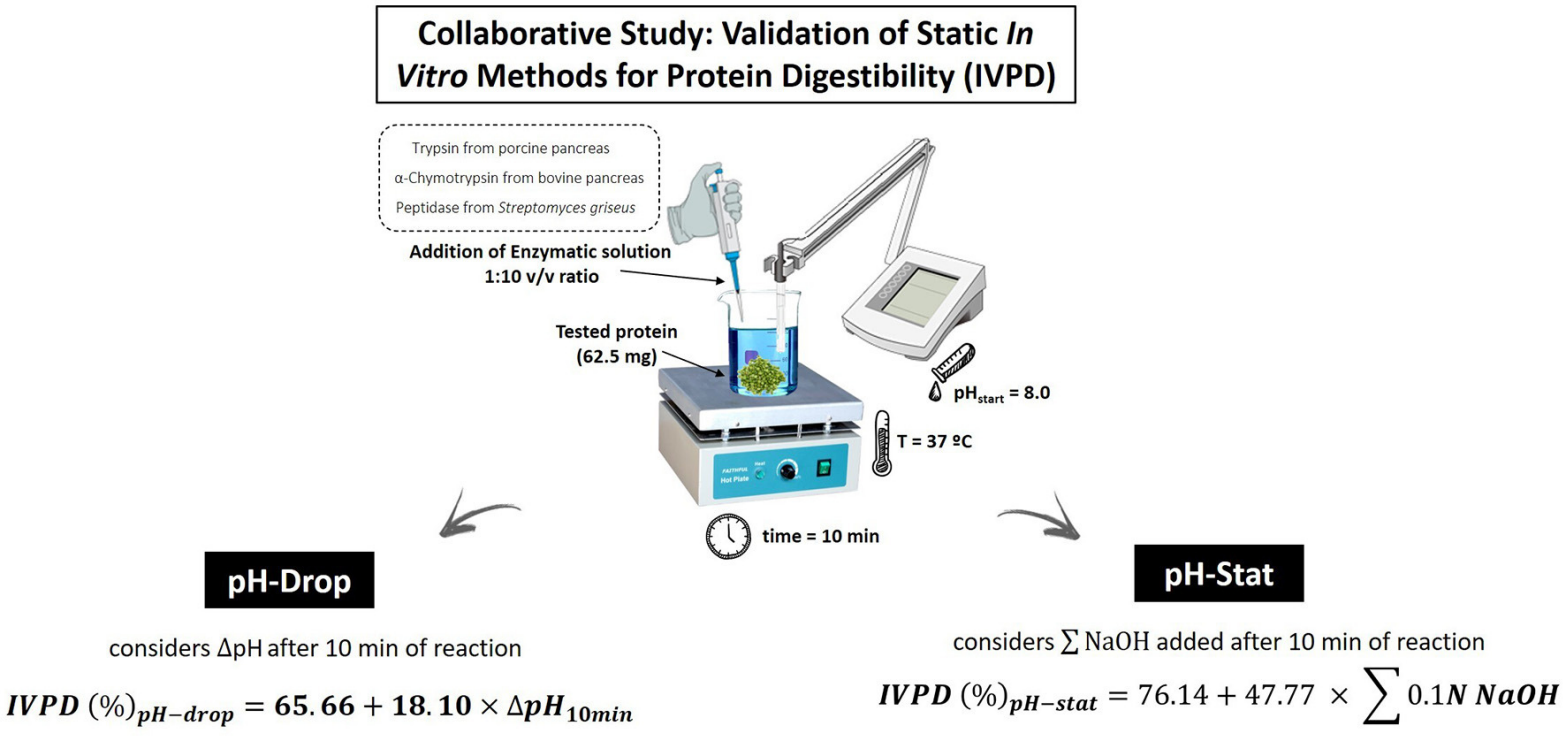
Schematic representation of static *in vitro* methods (pH-drop and pH-stat), included in a collaborative study aimed at validating their effectiveness in determining protein digestibility (47).

The FAO/WHO report (8) positioned two static, mono-compartmental models for measuring TFPD, namely the pH-drop (PHD) method (44, 45) and the pH-stat (PHS) method (46). These methods have been widely applied by researchers and digestibility measures show good agreement with *in vivo* protein digestibility (30). In general, both methods rely on the principle that as peptide bonds are cleaved during enzymatic digestion, protons are released and cause a drop in pH. Figure 1 represents a scheme of pH-drop and pH-stat analytical measurements for *in vitro* protein digestibility determination. The pH-drop method measures the drop in pH over a specified time, while the pH stat method maintains constant pH by auto-titration of NaOH. This method follows the pH change of the protein digestate over a 10-minute time period, and it has been shown to have a high correlation (R^2^ = 0.90) with the *in vivo* TFPD as determined in rats (44). A typical methodological approach includes taking 62.5 mg of protein equivalents (N × 6.25, standard nitrogen-to-protein conversion factor) from each test article for digestion. While it is recognized that 6.25 is not the appropriate nitrogen-to-protein conversion factor for all proteins, it is the default currently used until specific, validated and consensus-driven nitrogen-to-protein conversion determinations are established for all proteins (48). Test articles are digested by incubating them, in triplicate, with an enzyme cocktail containing 1.6 mg/ml trypsin [porcine pancreas 13,000–20,000 BAEE (Nα-benzoyl-L-arginine ethyl ester substrate) units/mg protein], 3.1 mg/ml chymotrypsin [bovine pancreas ≥40 N-Benzoyl-L- Tyrosine Ethyl Ester (BTEE) units/mg protein], and 1.3 mg/ml protease (*Streptomyces griseus* ≥3.5 units/mg solid) which are prepared in 10 ml of Milli-Q water and heated to 37°C. A modification of the original method was positioned by Tinus et al. (49) to account for changes in the availability of commercial proteases. The mixture is brought to a pH of 8.0 ± 0.5 with 1 M NaOH or HCl, after pH has stabilized following an hour of solubilization. The PHD is initialized with the addition of 1 ml of the enzymatic cocktail to the protein solution. The initial pH is recorded before the introduction of the cocktail and at 30 s intervals, for a total of 10 min. The PHD *in vitro* protein digestibility (IVPD, %) is calculated using the formula below:


$$\begin{array}{l} {IVPD\;\left(\%\right)}_{\;pH-drop}=65.66+18.10\;\times \;\Delta pH_{10min} \end{array}$$

The pH-stat assay, as mentioned, has generated protein digestibility values that agree well with *in vivo* measures with good reproducibility in an interlaboratory study (19). In brief, this method, which follows the protocol set out by Pedersen and Eggum (46), is similar to the pH-drop method. A typical approach involves the incubation of 62.5 mg of protein equivalents (N × 6.25) derived from test articles with an enzymatic cocktail containing 1.6 mg/ml trypsin, 3.1 mg/ml chymotrypsin, and 1.3 mg/ml protease, prepared in 10 ml of Milli-Q water and heated at 37°C. Both the sample protein and the enzyme cocktail are brought to a pH of 8.0 ± 0.5 with 1 M NaOH or HCl, following a 60 min pH stabilization process. Following the addition of enzymes, pH is held at 8.0 using 0.1 N NaOH, and the volume of 0.1 N NaOH used to hold the pH recorded. The pH-stat *in vitro* protein digestibility (IVPD, %) is calculated using the formula:


$$\begin{array}{l} {IVPD\;\left(\%\right)}_{\;pH-stat}=76.14+47.77\;\times \;\sum 0.1N\;NaOH \end{array}$$

One clear weakness of the pH-based assays is that foods with remarkably high buffering capacity, including some animal-based protein sources, may yield lower than expected digestibility values. In addition, the pH-based methods do not consider large intestinal fermentation that may contribute to the fecal nitrogen mass measured in the *in vivo* PDCAAS method. However, as indicated previously, the good agreement with published *in vitro* PDCAAS values to *in vivo* determined values suggests that the fecal nitrogen measurements do largely reflect the differences in small intestinal absorption of digestible amino acids from the food proteins. Therefore, the pH-based methods appear to closely calculate the digestible amino acids available for intestinal absorption. One additional limitation relates to the lack of use of pepsin (stomach protease) in these methods, however the use of a bacterial protease provides additional proteolytic activity to enhance overall protein digestibility. Despite these limitations, the documented agreement between the static methods and *in vivo* digestibility estimates supports their consideration for routine *in vitro* protein digestibility assessments for regulatory purposes. The simplicity, ease of implementation and low cost of executing these pH-based methods, however, warrants consideration and will be compared to a third method, the INFOGEST method, which, while modestly more complex, has been developed with the specific goal of standardizing experimental procedures and conditions for an *in vitro* digestion method that can be reproduced globally (50, 51).

### 3.2 Static, multi-compartment gastrointestinal digestion models

The INFOGEST network (http://www.cost-infogest.eu) was established in 2015 with the aim of “improving dissemination of critical research findings, developing truly multidisciplinary collaborations and harmonizing approaches between groups and discipline areas spanning the main stages of food digestion.” The network currently consists of more than 440 research scientists from 45 countries and includes 50 food companies.

The INFOGEST *in vitro* digestion method as originally developed (50) and as recently refined (INFOGEST 2.0) (51) is developed for food digestion in general and has been successfully used to assess protein digestibility in different foods and food forms (31, 52). This method is representative of gastrointestinal digestibility (GID) models. The INFOGEST method version 2.0 has been fine-tuned based on user feedback and precise and thorough details for the protocol have been published (51). The method is executed in three phases: preparation of the simulated digestive fluids and enzyme reagents, digestion procedure, and sample treatment for subsequent analyses (the latter being specific the specific assay endpoints) (51). The digestion procedure consists of three phases: (a) oral phase (salivary phase), (b) gastric phase, and (c) intestinal phase. Protein digestibility has been evaluated after INFOGEST digestion after each phase by arresting digestion. Different methods can be used to determine total protein digestibility, such as total nitrogen (e.g., Kjeldahl), primary amines (o-phtaldialdehyde, OPA) (53), trinitrobenzene sulfonic acid (TNBSA) (52, 54), and amino acid analysis (e.g., UPLC or LCMS) (55).

Harmonizing digestion techniques, exemplified by standardized INFOGEST protocols, holds a significant promise in advancing food digestion studies and crafting customized food solutions for diverse segments of the population (56, 57). However, while the static protocol improves comparability *in vivo* pig or rodent digestion, it does not fully capture dynamic *in vivo* digestion processes. Thus, direct comparison between *in vitro* and *in vivo* results remains crucial for validation (58, 59). Sousa et al. (55) evaluated the correlation between the *in vivo* and *in vitro* DIAAS, and results showed highly correlated true ileal digestibility values (*r* = 0.96, *R*^2^ = 0.89, *P* < 0.0001).

Furthermore, a patented method (U.S. Patent No. 9,738,920 B2) (60) outlines a technique for determining *in vitro* protein digestibility that involves a two-step enzymatic process. Initially, the protein-containing sample undergoes gastric digestion with the enzyme pepsin. Subsequently, the digested sample is treated with trypsin and chymotrypsin to simulate intestinal digestion. After these steps, a spectroscopic compound that binds with the protein's amino and carboxyl groups is added to create a solution suitable for optical analysis, such as the addition of ninhydrin, which produces Ruhemann's purple, and the absorbance is measured at 570 nm using a spectrophotometer. A commercial kit available from Megazyme contains all necessary enzymes and reagents for this procedure, allowing for the determination of the *in vitro* digestibility score.

## 4 Toward validation of *in vitro* methods for estimating protein digestibility for PDCAAS measurements

Despite the long-standing use of *in vitro* assays to determine protein digestibility, efforts to validate these methods as a replacement for the *in vivo* rat bioassay for the purposes of calculating PDCAAS have been limited. An interlaboratory study of the pH-stat *in vitro* method (46) for assessing protein digestibility was published in the same journal (61) just before a collaborative study on the *in vivo* method (using the same protein sources) was published. The latter led to the validation of the rat bioassay as an officially recognized method for protein digestibility (19). Notably, the reproducibility and repeatability of the *in vitro* and *in vivo* methods were similar, yet further action was not taken to promote the *in vitro* method presumably since *in vivo* methods were likely prioritized at the time. Additionally, while the pH-drop method has been used recently to determine the *in vitro* protein digestibility of a number of plant-based proteins, including pulses (33–36, 62), the method has been modified since first positioned (49), due principally to changes in the availability of key enzymes. As such, method validation remains a key goal for all of the static *in vitro* methods.

An important concept in developing and approving official methods of analyses is “fit for purpose” (30). The degree of accuracy and precision required for a measure of protein digestibility to enable a food product intended for consumption in mixed diets among the general public to carry a protein content claim must be such that it prevents overestimation of protein content, thereby avoiding the risk of underconsumption of indispensable amino acids. In countries and supranational unions, including the European Union and Australia (5), that do not require protein quality to be measured for protein content claims in foods intended for the general public (i.e., excluding special dietary uses), there have not been any reported safety issues or concerns of misleading consumers regarding choices of protein foods.

The current PDCAAS calculations have other sources of potential error which contribute to the value's inherent uncertainty, namely, the amino acid analyses (63, 64), total protein determination which depends on nitrogen determinations corrected for the protein-to-nitrogen conversion factor (for which there are no standardized factors) (48), and protein digestibility when using published tables of digestibility on a similar, but not the exact food, under study (65, 66). The latter source of error could be significantly reduced if a relatively inexpensive *in vitro*, high throughput, method of determining protein digestibility on the exact food and food forms were available. By convention, the reference protein, casein, used in many methods of assessing protein quality [i.e., Protein Efficiency Ratio (PER), Net Protein Utilization (NPU), and Indicator Amino Acid Oxidation (IAAO)] (30) would be chosen as the standard reference protein in the proposed collaborative study.

As mentioned above, the FAO/WHO has convened expert panels on numerous occasions to assess measures of protein quality and while PDCAAS remains the method of choice due to lack of data on emerging methodologies, recommendations to advance DIAAS were published in two key FAO reports published in 2013 (12) and 2014 (67). The DIAAS method uses the ileal digestibility coefficients of individual amino acids to determine the “true ileal digestibility” of the indispensable amino acids in food, unlike *in vivo* PDCAAS, which uses true fecal digestibility of the entire food protein for calculating protein quality values (68). While the DIAAS method may be a more accurate approach to determine protein quality (5, 13), the use of ileal-cannulated pigs as described is highly impractical to determine digestibility coefficients for large numbers of foods and food ingredients. As a result, several investigators working on standardizing the INFOGEST digestion method have recently published results that offer the promise of developing an *in vitro* DIAAS (IV-DIAAS) method (55, 69). Values for IV-DIAAS are comparable to that observed in the *in vivo* DIAAS method (69). A major ring-trial and methods validation protocol are currently underway for using INFOGEST 2.0 to determine *in vitro* DIAAS (ISO/NP 24167/IDF 261, Milk and milk products – *in vitro* digestion protocol for the analysis of protein digestibility and *in vitro* DIAAS). Supported by the International Dairy Federation (IDF), the joint IDF/International Standards Organization (ISO) *in vitro* protocol will first be applicable to dairy foods, with ring trial expansion to other foods, including plant-based proteins. The initial validation workflow was recently approved and the protocol has moved into a 36-month development track. While the *in vitro* DIAAS methodology can serve to generate proxy PDCAAS values (53), for the purposes of the remainder of this paper, the focus will be on the development of a collaborative study to determine *in vitro* TFPD for PDCAAS estimation and protein content claim substantiation.

Here, in order to address the major limitation of a lack of approved *in vitro* methods, we report that a collaborative study is ongoing that will evaluate and test the proficiency of candidate static *in vitro* methods to measure protein digestibility based on the currently accepted PDCAAS method. The primary objectives for this collaborative study are (a) to determine the repeatability and reproducibility of the candidate methods, and (b) to demonstrate sufficient agreement to published values of *in vivo* TFPD values to warrant approval as an official method of analysis and acceptance by regulatory bodies for protein quality assessments by PDCAAS. For the ongoing study, the focus will be on the pH-drop and the pH-stat methods, given their long-standing usage, available evidence to support agreement with *in vivo* TFPD data, and relative ease for implementation across multiple laboratory environments, including industry-based research settings.

## 5 Proposed pathway for validation for *in vitro* protein digestibility

The positioning of an approved *in vitro* method for assessing protein quality would provide an alternative to the use of animal bioassays for the substantiation of protein content claims. At present, protein remains the only nutrient for which the use of a bioassay is required to substantiate a protein content claim on foods in both Canada and the United States. Other nutrients, including energy, folate, niacin, and the fat-soluble vitamins, have established availability coefficients that have been accepted for labeling purposes, thus allowing analyzed food components to be converted to nutrient equivalents (e.g., dietary folate equivalent; Atwater factors for energy). Given that biological responses (PER; TFPD) are required for protein, this creates barriers for the food sector to differentiate both existing and new protein sources in terms of their ability to contribute quality protein for the human diet. The acceptance of an approved *in vitro* method for estimating TFPD would address this challenge. It would provide regulatory agencies assurances that factors influencing the digestibility of dietary proteins, particularly new sources and those derived from new processing methods, have been considered. Additionally, the acceptance of one (or more) approved methods would provide conformity within the food system as to the methods to use when positioning food protein sources for human consumers.

**Figure 2:**
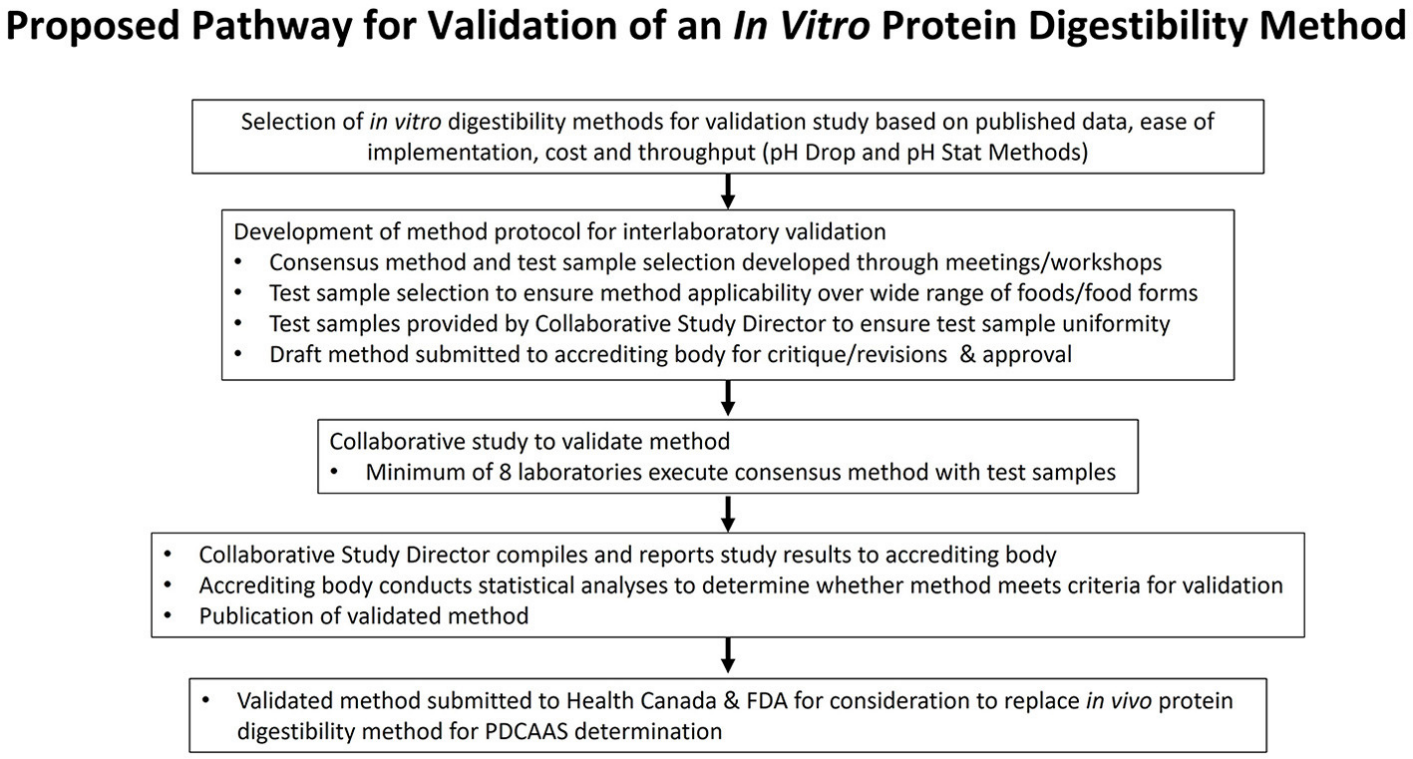
Proposed pathway for validation of an *in vitro* protein digestibility method.

In order to position an *in vitro* method to both Health Canada and the FDA as being a suitable substitute for the TFPD bioassay, the method must first be approved by an accrediting body. Such bodies include the Association of Official Analytical Collaboration (AOAC), the American Oil Chemists' Society (AOCS) or International Organization for Standardization (ISO). The methods approval process employed by authoritative bodies typically involves a series of sequential steps that begin with the submission of a proposed method, either on its own or accompanied by results from a collaborative study. This process allows for an initial review of the method by the certifying body, providing a chance for experts to offer commentary and feedback on the proposal before it undergoes a collaborative study. As such, this approach can mitigate risks associated with methodological concerns prior to the initiation of data generation. Once the method has been approved by the sub-committee, a collaborative study is conducted to generate the data that will then be reviewed by a separate statistical sub-committee enroute to subsequent approval steps. Key to this process is the positioning of a method that has been written and structured according to the style guidance of the approving body. Figure 2 represents the proposed pathway for validation of an *in vitro* protein digestibility method. Regulatory bodies usually mandate the use of approved official methods to meet their scientific requirements for labeling, therefore once the *in vitro* digestibility method is approved as an official method by an accrediting body, the final step would be to petition the appropriate regulatory bodies to accept the official method for PDCAAS calculations.

The following components represent the required elements of an AOCS official method: (1) title of the method; (2) definition of the method including a description of the analyte or component in question; (3) scope of the method, including a description of the test articles to which the method applies; (4) apparatus to be used in the method; (5) reagents to be used, including information on the reagent grade and sourcing as well as pertinent information on the usage of special solutions; (6) procedural information to provide clear instructions for the analysis in question; (7) calculations required for the proposed method, presented in sufficient detail; (8) precision data derived from a collaborative study to support the method; (9) notes that pertain to the method, including safety concerns, data on limits of detection and other comments that are pertinent; and (10) key references, tables and figures.

As the static *in vitro* methods have been published, the methods for both the pH-drop and pH-stat have been based on those key publications (44, 46), with subsequent modifications (49). The draft test methods for both approaches have been submitted to potential study collaborators and consensus on the specific methods has been achieved through a workshop held in the spring of 2023. The Richardson Center for Food Technology and Research is serving as the Central Laboratory and we have successfully recruited a minimum of eight laboratories for the ring test as typically required by authoritative bodies for the generation of data for precision, repeatability, reproducibility, and accuracy. This standard ensures a broad and reliable data set for method validation. Key considerations for establishing a method that could be readily adopted and accepted by regulators include the availability of method reagents and apparatus. For *in vitro* protein digestibility (IVPD) determinations, changes in availability of enzymes, for example, will require standardization and consensus on suitable alternatives. The consensus method has been submitted and approved by Uniform Methods Committee of the AOCS. The proficiency testing began in the summer of 2023 and once results from all participating laboratories has been received, the data will be analyzed according to standard statistical practices for collaborative studies. Once feedback has been received from the approving organization, the methods would be shared with the regulatory bodies for commentary and critique, with further method refinement as needed in order to gain acceptance for the *in vitro* methods in PDCAAS calculations.

A key consideration for advancing the methods is the establishment of data across a number of test protein sources. The choice of protein food samples was critical for this evaluation to ensure applicability over a wide range of foods and food forms, including plant, animal, and novel protein sources. Casein was included as the standard comparator (as it is the standard in the current official *in vivo* method). A slate of test protein sources determined by consensus among the participating laboratories is positioned in Table 1. The test protein sources were selected to represent a range of plant and animal proteins and levels of processing, and to reflect those samples for which TFPD values are generally available. The latter will be an important criterion for providing evidence to the regulatory authorities on the validity of the generated *in vitro* data for estimating *in vivo* TFPD values. To facilitate the collaborative study, the central laboratory is responsible for the procurement, processing, packaging and distribution of test articles to the collaborating laboratories, as well as the collection of the sample data and statistical analysis in advance of submission to the approving body. During the course of this work and as mentioned above, consultation with the approving body and representatives from the FDA and Health Canada is ongoing and will be critical to ensure that criteria required for regulatory acceptance of the *in vitro* methods are addressed prior to completing the collaborative study execution and analyses.

**Table 1:** Potential protein test articles for the validation of *in vitro* methods for the estimation of true fecal protein digestibility.

**Source**	**Forms**	**% True fecal protein digestibility**	**References**
Casein	As procured	96–99	(8, 65)
Egg white powder	As procured	97–98	(65)
Skim milk powder	As procured	95	(8)
Beef, ground	Cooked; dried	91–95	(65)
Yellow pea	Thermal treatment; isolate	86–89 (cooked)	(33, 65)
Bean (pinto)	Thermal treatment	63 (baked); 76 (boiled); 85 (extruded)	(34)
Green lentil	Thermal treatment	86–88 (boiled); 86 (extruded)	(36)
Soy	Autoclaved; flour; isolate	84 (flour); 95 (concentrate); 96 (isolate)	(65)
Potato	Raw; boiled; isolate	40 (raw); 83 (boiled)	(70)
Rice	Raw; cooked; isolate	87 (polished); 86 (cooked)	(8)
Wheat	Raw; flour; gluten	87 (whole); 97 (white flour); 98 (gluten)	(65)

## 6 Conclusions

The adoption of validated *in vitro* methods to measure protein digestibility (IVPD) for Protein Digestibility Corrected Amino Acid Score (PDCAAS) assessments will pave the way for innovation in the production and marketing of protein-rich foods in Canada and the United States. This regulatory change, eliminating the reliance on animal-based bioassays to confirm protein quality, is supported by substantial evidence endorsing *in vitro* methods capable of distinguishing between low and high-quality protein sources (18). This report outlines the steps for evaluating potential *in vitro* protein digestibility methods through an ongoing collaborative study, aiming to achieve certification from an authoritative body. Through a continuous and collaborative approach, the ultimate objective is to gain approval for one or more *in vitro* methods for determining protein digestibility in PDCAAS calculations, thereby marking the success of this initiative within North America. Furthermore, it is important to consider the global leadership role that the United States and Canada can play in shaping international standards for protein quality assessment. While the focus has primarily been on the regulatory implications within North America, it is crucial to acknowledge broader perspectives and future directions. Although PDCAAS has been widely used, the limitations associated with certain protein sources underscore the need for considering alternative measures, such as DIAAS, which offer a more precise assessment of protein quality but still currently requires *in vivo* assessment of protein digestibility. The efforts in advancing *in vitro* methods could serve as a model where *in vivo* methods are still required for certain protein foods, contributing to harmonized regulatory frameworks and facilitating international trade of protein-rich foods. In moving forward, it is essential to consider the complexities of inter-individual variability in protein digestibility and the influence of food matrices on digestibility assessments. Future research should explore the applicability of these methods in real food matrices and address population-specific variations in protein digestion.

## Author contributions

EK: Conceptualization, Formal analysis, Project administration, Writing – original draft. AS: Validation, Visualization, Writing – review & editing. EG: Validation, Visualization, Writing – review & editing. JH: Supervision, Conceptualization, Project administration, Resources, Writing – review & editing, Funding acquisition.
